# Carotid Plaque-RADS improves preoperative coronary risk stratification in candidates for carotid revascularization

**DOI:** 10.1186/s13244-025-02188-y

**Published:** 2026-01-26

**Authors:** Rui Qin, Chong Zheng, Yue Zhang, Mengmeng Feng, Senhao Zhang, Qun Gai, Zihang Liu, Tong Li, Ximing Wang, Jie Lu

**Affiliations:** 1 Department of Radiology and Nuclear Medicine, Xuanwu Hospital, Capital Medical University, Changchun Street, No. 45, 100053 Beijing, China; 2 Beijing Key Laboratory of Magnetic Resonance Imaging and Brain Informatics, 100053 Beijing, China; 3 Central Research Institute, United Imaging Healthcare, Shanghai, China; 4 Department of Radiology, Shandong Provincial Hospital Affiliated to Shandong First Medical University, Jinan, China

**Keywords:** Carotid atherosclerosis, Coronary artery stenosis, High-resolution magnetic resonance imaging, Computed tomography, Fractional flow reserve

## Abstract

**Objectives:**

In this retrospective study, we aimed to assess the predictive value of the Carotid Plaque-RADS (Reporting and Data System) for coronary functional stenosis in candidates for carotid revascularization, using high-resolution magnetic resonance imaging (HR-MRI) coupled with computed tomography-derived fractional flow reserve (CT-FFR).

**Materials and methods:**

A retrospective analysis was performed on data of 101 patients with carotid atherosclerosis who underwent HR-MRI for Carotid Plaque evaluation, and CT-FFR for coronary assessment was conducted. Patients were divided into two groups based on a CT-FFR threshold of ≤ 0.80. Logistic regression, correlation analyses, and receiver operating characteristic curve analyses were used to identify predictors of coronary functional stenosis.

**Results:**

In the functional stenosis group (*n* = 76), both plaque volume and Carotid Plaque-RADS categories had higher values than those observed in the non-functional group (*n* = 25). Univariate analysis showed that Carotid Plaque-RADS, Carotid Plaque volume, and hypertension were associated with functional stenosis. After adjustment, Carotid Plaque-RADS remained an independent predictor (odds ratio: 2.35, *p* < 0.01) and demonstrated the strongest correlation (*ρ* = 0.51, *p* < 0.01). It also demonstrated good diagnostic performance (area under the curve [AUC]: 0.81; sensitivity: 85%; specificity: 68%) and favorable clinical utility on decision curve analysis. In an exploratory analysis, Carotid Plaque-RADS was also moderately correlated with CAD-RADS (*ρ* = 0.37, *p* < 0.01) and predicted CAD-RADS ≥ 3 with good discrimination (AUC: 0.72).

**Conclusion:**

Carotid Plaque-RADS is an independent, noninvasive predictor of coronary functional stenosis in candidates for carotid revascularization.

**Critical relevance statement:**

Carotid Plaque-RADS provides a noninvasive imaging-based tool that independently predicts coronary functional stenosis, thereby enhancing preoperative coronary risk stratification and supporting integrated cardiovascular management in candidates for carotid revascularization.

**Key Points:**

- Carotid revascularization candidates face high coronary risk.
- Carotid Plaque-RADS independently predicts coronary functional stenosis.
- Carotid Plaque-RADS enhances preoperative coronary risk stratification.

**Graphical Abstract:**

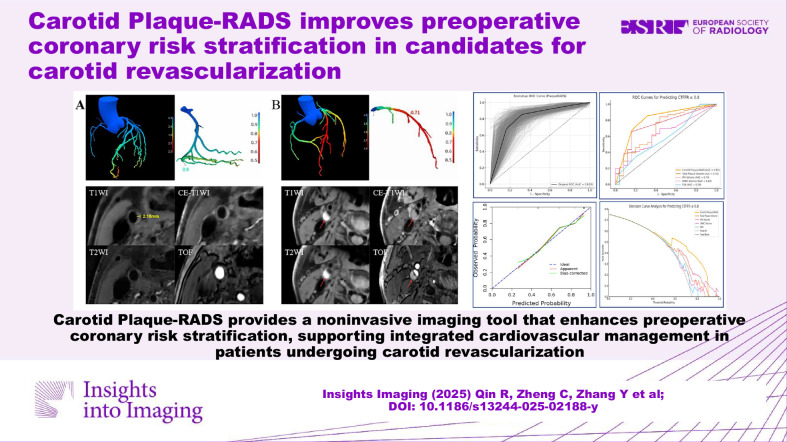

## Introduction

Atherosclerosis is a systemic disease that commonly involves multiple vascular territories, particularly the carotid and coronary arteries [1, 2]. Increasing evidence shows that plaque characteristics in one vascular bed often mirror those in others, highlighting the systemic nature of the disease and its relevance for cardiovascular risk stratification [3, 4]. Patients with multivessel atherosclerosis, especially those with concurrent carotid and coronary artery disease (CAD), are more likely to develop adverse cardiovascular events and recurrence compared to those with single-vessel disease [5–7]. Early identification of such high-risk individuals is critical for optimizing management. Patients undergoing evaluation for carotid revascularization represent a particularly high-risk population, as many have undiagnosed CAD that increases perioperative cardiovascular risk, while routine invasive coronary testing is often impractical. This underscores the need for reliable, noninvasive tools to identify coronary disease with functional significance and guide perioperative and long-term management [8]. Given this clinical need, a retrospective study was conducted to investigate whether Carotid Plaque characteristics can predict coronary functional stenosis.

Coronary computed tomography-derived fractional flow reserve (CT-FFR) is a noninvasive technique to assess the functional significance of coronary stenosis, with a CT-FFR ≤ 0.80 widely accepted to indicate functional impairment [9, 10]. Although CT-FFR provides a functional evaluation of coronary stenosis, it remains unclear whether carotid imaging biomarkers can serve as surrogate indicators for identifying patients with functionally significant CAD.

Traditionally, carotid imaging has focused on luminal narrowing. However, emerging evidence suggests that plaque composition and vulnerability are stronger predictors of cardiovascular risk than stenosis severity alone [11, 12]. High-resolution magnetic resonance imaging (HR-MRI) excels in precisely evaluating elements such as plaque components of intraplaque hemorrhage (IPH), lipid-rich necrotic core (LRNC), and calcification, which can increase the risk of plaque rupture and ischemic complications [13]. To standardize plaque assessment, the Carotid Plaque-RADS (Reporting and Data System) was recently introduced, providing a structured framework to evaluate plaque morphology and risk based on imaging characteristics [14].

Although Carotid Plaque-RADS shows promise for cerebrovascular risk stratification [15–17], its application in assessing coronary risk among high-risk surgical candidates has not been well-established. Therefore, we aimed to assess the predictive value of Carotid Plaque-RADS for identifying functional coronary stenosis in patients undergoing evaluation for carotid revascularization. In addition, we explored the relationship between Carotid Plaque-RADS and CAD-RADS to provide an anatomical cross-validation of the systemic atherosclerotic burden.

## Materials and methods

### Study participants

In this retrospective study, we included patients with carotid atherosclerotic plaques who were referred for preoperative vascular evaluation for potential carotid revascularization between 2019 and 2024 at Xuanwu Hospital or Shandong Provincial Hospital. Patients were deemed eligible for carotid revascularization according to established clinical guidelines, including symptomatic patients with ≥ 50% carotid stenosis and asymptomatic patients with ≥ 70% stenosis, as measured using duplex ultrasound or carotid HR-MRI. All patients underwent HR-MRI as part of their preoperative workup, regardless of whether carotid intervention was ultimately performed. Coronary computed tomography angiography (CCTA) was performed on all patients during hospitalization. This study was approved by the Ethics Committee of Xuanwu Hospital, Capital Medical University (approval no.: KS2022023-1).

The inclusion criteria were as follows: (1) patients undergoing preoperative evaluation for carotid revascularization evaluation based on symptoms and imaging; (2) presence of carotid atherosclerosis confirmed using HR-MRI; (3) HR-MRI, CCTA, and relevant laboratory tests performed within 2 weeks; and (4) availability of complete clinical data.

The exclusion criteria were the following: (1) incomplete or poor-quality HR-MRI or CCTA images; (2) non-atherosclerotic vascular diseases, such as carotid dissection, fibromuscular dysplasia, arteritis, or non-atherosclerotic coronary diseases; and (3) acute cardiovascular or cerebrovascular events; (4) prior history of coronary revascularization (percutaneous coronary intervention or coronary artery bypass grafting); (5) previous history of carotid artery interventions (carotid endarterectomy or stenting); and (6) history of malignancy or prior neck radiotherapy.

### Clinical data collection

Patient data were collected from electronic health records, including age, sex, hypertension, diabetes mellitus, dyslipidemia, smoking status, alcohol consumption, systolic and diastolic blood pressure, total cholesterol (TC), triglycerides, high- (HDL-C) and low-density lipoprotein cholesterol (LDL-C), apolipoprotein A, apolipoprotein B, homocysteine, fibrinogen, symptomatic carotid stenosis, and Framingham risk score (FRS). The FRS was calculated based on established criteria, incorporating age, sex, TC, HDL-C, smoking status, blood pressure, and diabetes status [18].

### Image acquisition

#### Carotid HR-MRI acquisition

HR-MRI of the carotid arteries was performed using a 3.0-T PET/MR system (uPMR790, United Imaging, Xuanwu Hospital, Capital Medical University) and a 3.0-T MRI system (Siemens Prisma, Shandong Provincial Hospital). Patients were positioned supine with the chin slightly elevated to optimize visualization of the bilateral carotid arteries. Imaging was performed using a dedicated head-neck coil in combination with an eight-channel surface coil for carotid artery imaging. The surface coil was securely fixed to minimize motion artifacts and ensure consistent image quality. All patients underwent multi-sequence imaging, including T1-weighted imaging (T1WI), T2-weighted imaging (T2WI), three-dimensional time-of-flight (3D-TOF) magnetic resonance angiography, and contrast-enhanced T1WI (CE-T1WI). Further information is available in the Supplemental Methods.

#### CCTA acquisition

CCTA was performed using either a dual-source CT scanner (SOMATOM Definition Flash/Force; Siemens Healthineers) or a 256-slice CT scanner (Revolution CT; GE Healthcare). For Siemens scanners, retrospective ECG-gated spiral acquisition was used (90/100 kVp, 370  mAs, CARE Dose 4D, rotation time 0.28/0.25 s), with a slice thickness of 0.75 mm, 0.5 mm interval, and a medium soft-tissue kernel (I26f/Bv40). For the GE scanners, prospective ECG-triggered axial acquisition was employed (100 kVp, 350–650 mA, noise index 20 HU, rotation time 0.28 s), with the same slice thickness and increment using hybrid iterative reconstruction (ASiR-Veo) with 50% blending. In both protocols, iodixanol (350 mgI/mL) was administered at 30.0–55.0 mL and 3.5–5.5 mL/s, followed by a saline flush. Bolus tracking was used, with scanning initiated 5 s after aortic root enhancement reached 100 HU.

### Image analysis

#### Carotid HR-MRI analysis

Two blinded radiologists independently reviewed all carotid HR-MRI examinations. Both had substantial experience in vascular imaging (10 and 15 years, respectively). Any discrepancies were resolved by consensus. Carotid stenosis was quantified as the ratio of the minimum lumen diameter to that of the adjacent normal segment, and categorized as mild (< 50%), moderate (50–69%), severe (70–99%), or occlusion [19].

Plaque signal intensity was referenced to the adjacent sternocleidomastoid muscle. IPH was identified by a high signal on TOF and T1WI, with variable signal on T2WI depending on the age of the hemorrhage [20]. LRNC appeared isointense or slightly hyperintense on TOF and T1WI, isointense or hypointense on T2WI, and hypointense on CE-T1WI [20]. Calcifications were hypointense across all sequences [21]. Total plaque, IPH, and LRNC volumes were measured using MRI-Plaque View software [22].

Plaque evaluation was performed using Carotid Plaque-RADS, providing a standardized imaging-based assessment of plaques by morphology and associated cardiovascular risk [14]: Carotid Plaque-RADS 2, maximum wall thickness (MWT) < 3 mm; Carotid Plaque-RADS 3a, LRNC with intact thick fibrous cap (MWT ≥ 3 mm); Carotid Plaque-RADS 3b, LRNC with thin fibrous cap (MWT ≥ 3 mm); Carotid Plaque-RADS 3c, healed ulcerated plaques (MWT ≥ 3 mm); and Carotid Plaque-RADS 4, high-risk features, including those with a complicated morphology, IPH, ruptured fibrous caps, and intraluminal thrombi.

#### CCTA analysis

Two blinded radiologists independently reviewed all CCTA examinations. Both had substantial experience in vascular imaging (10 and 15 years, respectively). Any discrepancies were resolved by consensus. CT-FFR was assessed using an artificial intelligence-based computational fluid dynamics algorithm (SkCT-FFR, Beijing, China), integrating anatomical and hemodynamic modeling noninvasively [23]. The CT-FFR analysis was performed using a dedicated workstation with automatic image preprocessing, segmentation, and hemodynamic computations. For vessels with stenosis, CT-FFR was measured 2 cm distal to the segment with the most severe stenosis; for vessels without stenosis, measurement was taken at the point where the vessel diameter reached 1.5 mm. A CT-FFR value ≤ 0.80 was considered functionally significant. All results were reviewed by experienced radiologists to ensure image quality and accuracy. Coronary stenosis severity was assessed on CCTA using the CAD-RADS classification in accordance with the CAD-RADS 2.0 consensus [24].

#### Statistical evaluation

Statistical analyses were conducted using SPSS version 27 (IBM Corp.) and R version 4.4.1. Continuous variables were tested for normality using the Kolmogorov–Smirnov test and expressed as mean ± standard deviation or median (interquartile range). Categorical variables are presented as frequencies and percentages. Continuous variables were compared between the non-functional and functional stenosis groups using the independent-samples *t*-test or Mann–Whitney *U*-test, as appropriate, while categorical variables were compared using the chi-square test or Fisher’s exact test.

Spearman’s correlation analysis was used to assess associations between continuous and ordinal variables, whereas phi coefficients were calculated for binary variables. Univariate logistic regression analysis was used to identify potential clinical and imaging predictors of coronary functional stenosis. Variables with *p* < 0.05 were included in a multivariate logistic regression analysis using the Enter method to determine independent predictors, with odds ratios (ORs) and 95% confidence intervals (CIs) reported.

The diagnostic performance of Carotid Plaque-RADS and other predictors was assessed using receiver operating characteristic (ROC) curve analysis, with the area under the curve (AUC), sensitivity, specificity, and accuracy calculated. Model stability was evaluated using 1,000 bootstrap resamples. The Hosmer–Lemeshow goodness-of-fit test was employed to assess model calibration, with calibration curves plotted accordingly. Comparisons of AUCs between different predictors were performed using the DeLong test. Decision curve analysis (DCA) was conducted to evaluate clinical utility and net benefit across varying thresholds. A two-tailed *p *< 0.05 was considered statistically significant.

## Results

### Clinical characteristics

**Fig. 1:**
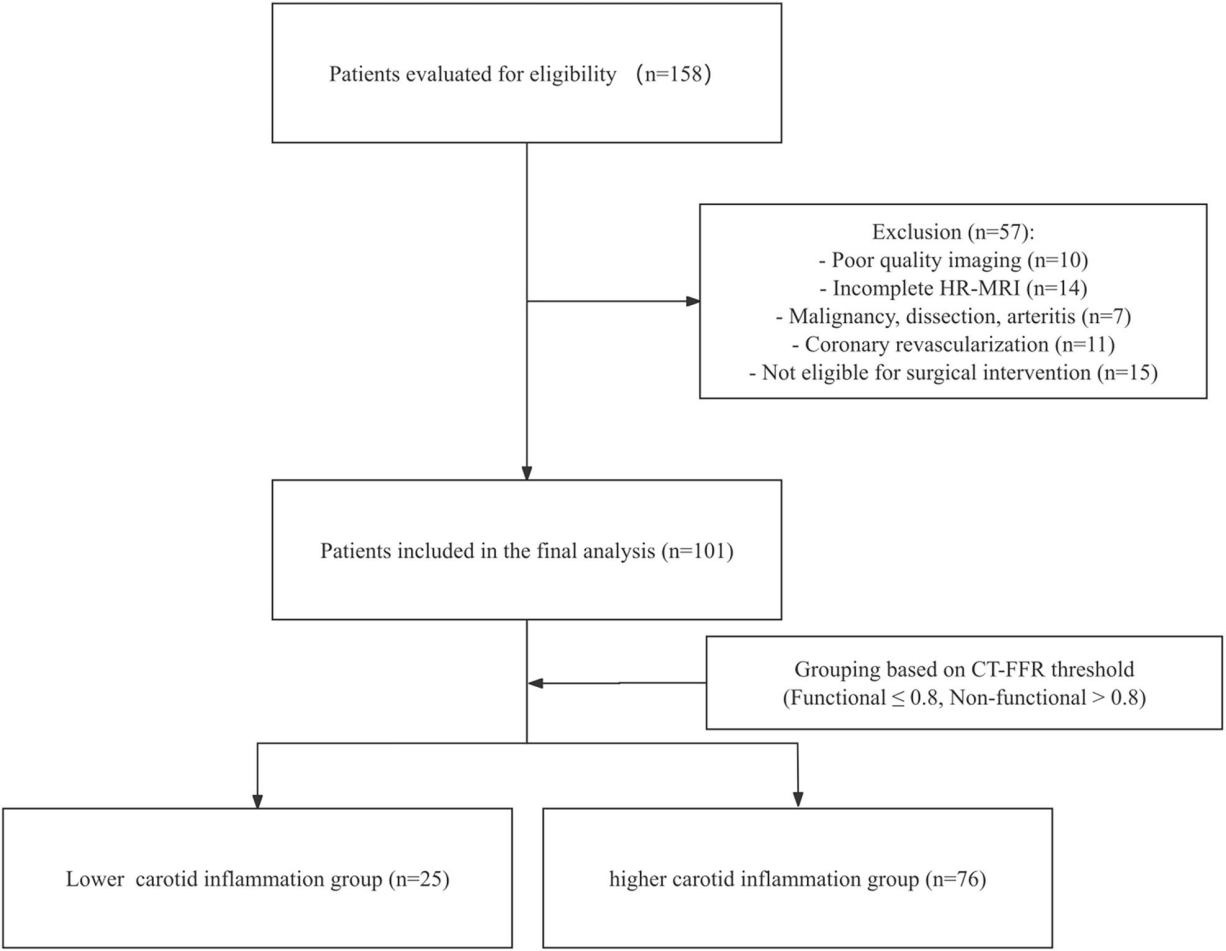
Flowchart of patient selection and grouping based on CT-FFR assessment. CT-FFR, computed tomography-derived fractional flow reserve; HR-MRI, high-resolution magnetic resonance imaging

After applying these criteria, 158 patients were excluded for the following reasons: inadequate image quality (*n* = 10); incomplete HR-MRI sequences (*n* = 14); malignancy or non-atherosclerotic vascular disease such as carotid dissection, or arteritis (*n* = 7); prior coronary revascularization (*n* = 11); and not meeting clinical indications for carotid revascularization (*n* = 15). Ultimately, 101 patients were included in the final analysis (Fig. 1). The baseline clinical characteristics of the non-functional (*n* = 25) and functional (*n* = 76) stenosis groups are listed in Table 1. Variables such as age, sex, diabetes mellitus, dyslipidemia, smoking status, drinking status, and TC were not significantly different (all *p* > 0.05). Medication use, including statins, antihypertensive agents, and antiplatelet therapy, was also recorded and compared between groups, with no significant differences observed (all *p* > 0.05). However, the functional stenosis group had a significantly higher prevalence of hypertension compared with the non-functional stenosis group (72.37% vs. 48%, *p* = 0.02).

**Table 1:** Comparison of clinical characteristics between non-functional and functional stenosis groups

Clinical characteristics	Non-functional stenosis group (*n* = 25)	Functional stenosis group (*n* = 76)	*T*/*Z*/*χ*^2^	*p*-value
Age, years	67.12 ± 8.21	64.93 ± 7.09	1.28	0.20
Male, No. (%)	20 (80.00)	64 (84.21)	0.23	0.62
Hypertension, no. (%)	12 (48.00)	55 (72.37)	0.43	0.02*
Diabetes mellitus, no. (%)	7 (28.00)	13 (17.11)	1.40	0.23
Dyslipidemia, no. (%)	7 (28.60)	26 (34.21)	0.33	0.56
Current smokers, no. (%)	11 (44.00)	47 (61.84)	2.24	0.11
Current drinkers, no. (%)	10 (40.00)	42 (55.26)	1.75	0.18
Total cholesterol, mmol/L	3.98 ± 0.95	3.70 ± 1.14	1.10	0.27
Triglycerides, mmol/L	1.33 (0.8, 1.7)	1.39 (1.0,1.8)	−1.27	0.20
HDL-C, mmol/L	1.22 (1.0, 1.4)	1.04 (0.9,1.3)	−1.78	0.07
LDL-C, mmol/L	2.20 (1.7, 3.1)	1.95 (1.6,2.6)	−1.04	0.29
Apolipoprotein A, g/L	1.14 (1.0, 1.2)	1.13 (1.0,1.3)	−0.02	0.98
Apolipoprotein B, g/L	0.75 (0.7, 1.0)	0.75 (0.6,0.9)	−0.57	0.56
Homocysteine, umol/L	13.63 (11.8, 14.7)	14.90 (11.1, 17.7)	−0.74	0.45
Fibrinogen, g/L	3.23 ± 0.68	3.46 ± 0.71	−1.41	0.16
Framingham risk score	14.00 (13.0, 15.0)	15.00 (13.0, 16.0)	−1.40	0.16
Antihypertensive drug, no. (%)	13 (52.00)	49 (64.50)	1.23	0.27
Statins, no. (%)	18 (72.00)	58 (76.30)	0.19	0.66
Antiplatelet drug, no. (%)	17 (68.00)	54 (71.10)	0.08	0.77

*HDL-C* high-density lipoprotein cholesterol, *LDL-C* low-density lipoprotein cholesterol

* *p* < 0.05

### Carotid artery characteristics

**Fig. 2:**
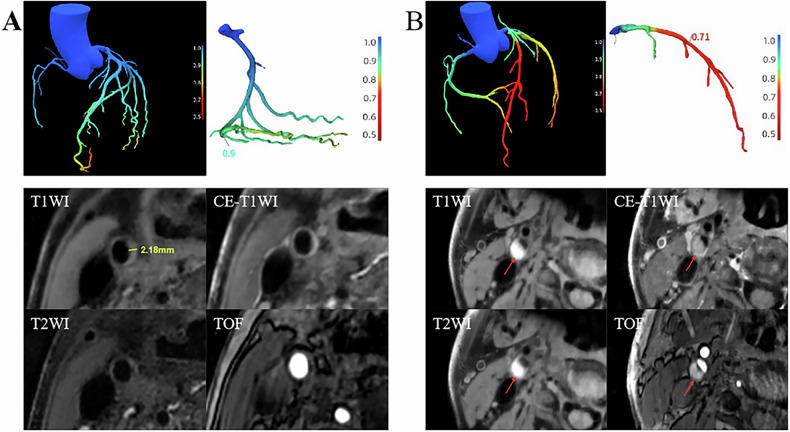
Carotid plaque imaging features in patients stratified by the presence of functional coronary artery stenosis. **A** A patient without coronary functional stenosis, whose lowest CT-FFR value is 0.90. The carotid artery wall thickness is 2.18 mm, which is less than 3 mm, and there are no features of complicated plaque or ulcerations. As a result, the patient’s Carotid Plaque-RADS is classified as level 2. **B** A patient with coronary functional stenosis, whose lowest CT-FFR value is 0.71. Intraplaque hemorrhage (indicated by arrows) is observed in the right internal carotid artery, and the patient’s Carotid Plaque-RADS is classified as level 4. T1WI, T1-weighted imaging; CE-T1WI, contrast-enhanced T1-weighted imaging; T2WI, T2-weighted imaging; TOF, time-of-flight imaging

Table 2 and Fig. 2 show the differences in carotid stenosis severity, plaque classification, and composition between the two groups. The functional stenosis group exhibited a significantly different distribution of Carotid Plaque-RADS categories (*p* < 0.01) compared with the non-functional stenosis group. Additionally, this group had significantly higher volumes of IPH, LRNC, and total plaque (all *p* < 0.01). In contrast, carotid stenosis severity and plaque calcification did not differ significantly between the two groups (both *p *> 0.05).

**Table 2:** Comparison of carotid artery characteristics between the non-functional and functional stenosis groups

Characteristics	Non-functional stenosis group (*n* = 25)	Functional stenosis group (*n* = 76)	*Z*/*χ*^2^	*p*-value
Carotid stenosis degree No. (%)			0.67	0.71
2 (50–69%)	7 (28.00)	17 (22.37)		
3 (70–99%)	16 (64.00)	55 (72.37)		
4 (Occlusion)	2 (8)	4 (5.26)		
Carotid Plaque-RADS, No. (%)			28.69	< 0.01^**^
2	4 (16)	2 (2.63)		
3a	13 (52.00)	9 (11.84)		
3b	3 (12.00)	10 (13.16)		
3c	1 (4.00)	4 (5.26)		
4	4 (16.00)	51 (67.11)		
IPH volume, mm^3^	0 (0, 0)	87.21 (0.00, 339.80)	−3.82	< 0.01^**^
LRNC volume, mm^3^	29.01 (0.00, 60.56)	112.30 (0.00, 256.60)	−1.98	0.04^*^
Total plaque volume, mm^3^	499.60 (306.70, 1127)	1071 (630.90, 1487.50)	−3.11	< 0.01^**^
Calcification, no. (%)	13 (52.00)	54 (71.05)	3.05	0.08

*IPH* intraplaque hemorrhage, *LRNC* lipid-rich necrotic core, *RADS* Reporting and Data System

* *p* < 0.05, ** *p* < 0.01

### Correlation analysis

Spearman correlation analysis demonstrated that the Carotid Plaque-RADS was most strongly correlated with coronary functional stenosis (*ρ* = 0.51, *p* < 0.01). The total plaque (*ρ* = 0.29, *p* < 0.01), IPH (*ρ* = 0.38, *p* < 0.01), and LRNC (*ρ* = 0.20, *p* = 0.04) volumes also showed significant positive correlations. Among binary variables, hypertension (*φ* = 0.20, *p* = 0.04) was significantly associated with functional stenosis, indicating its potential role as a contributing risk factor. In an additional analysis, Carotid Plaque-RADS was moderately correlated with coronary anatomical stenosis, as assessed by CAD-RADS (*ρ* = 0.37, *p* < 0.01).

### Logistic regression analysis of risk factors for coronary functional stenosis

Univariate logistic regression analysis identified several significant predictors of functional stenosis, including hypertension (OR: 2.84, *p* = 0.02), total plaque volume (OR: 1.01, *p* = 0.02), IPH volume (OR: 1.01, *p* = 0.02), LRNC volume (OR: 1.01, *p* = 0.04), and Carotid Plaque-RADS (OR: 2.47, *p *< 0.01). In multivariate analysis adjusted for confounders, only the Carotid Plaque-RADS remained an independent predictor of functional stenosis (OR: 2.35, *p* < 0.01). Relevant results are summarized in Table 3.

**Table 3:** Logistic regression analysis of clinical and plaque-related risk factors for coronary functional stenosis

Variables	Univariate logistic regression						Multivariate logistic regression				
	*β*	S.E	*Z*	*p*	OR (95% CI)		*β*	S.E	*Z*	*p*	OR (95% CI)
Hypertension	1.04	0.48	2.19	0.02*	2.84 (1.12–7.20)		1.04	0.59	1.77	0.07	2.84 (0.89–9.05)
Total plaque volume	0.01	0.00	2.37	0.02*	1.01 (1.01–1.01)		−0.00	0.00	-0.68	0.49	1.00 (1.00–1.00)
IPH volume	0.01	0.00	2.37	0.02*	1.01 (1.01–1.01)		0.00	0.00	0.39	0.69	1.00 (1.00–1.00)
LRNC volume	0.01	0.00	2.06	0.04*	1.01 (1.01–1.01)		0.00	0.00	0.90	0.37	1.00 (1.00–1.01)
Carotid Plaque-RADS	0.90	0.20	4.54	< 0.01**	2.47 (1.67–3.64)		0.85	0.27	3.21	< 0.01**	2.35 (1.39–3.96)

*OR* odds ratio, *CI* confidence interval, *FRS* Framingham risk score, *HDL-C* high-density lipoprotein cholesterol, *IPH* intraplaque hemorrhage, *LRNC* lipid-rich necrotic core, *RADS* Reporting and Data System

* *p* < 0.05, ** *p* < 0.01

### Diagnostic performance of Carotid Plaque-RADS

**Fig. 3:**
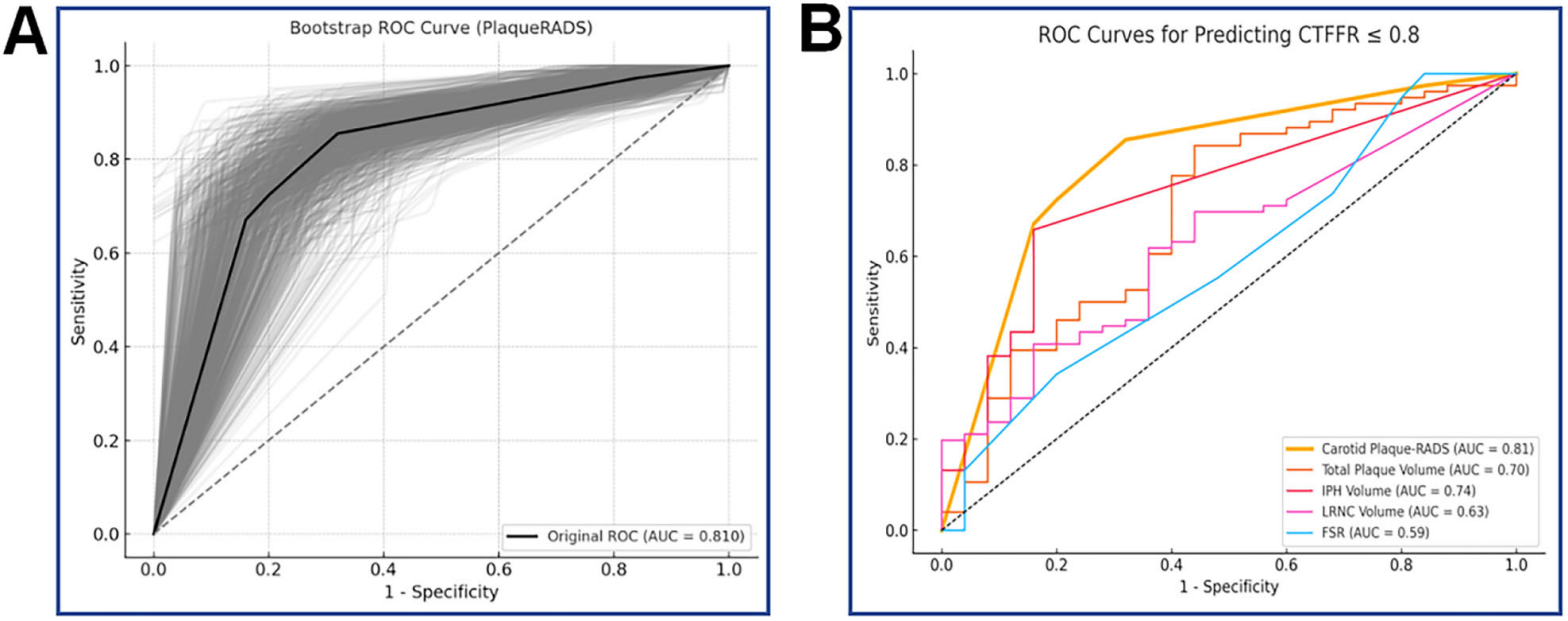
Diagnostic performance of Carotid Plaque-RADS and other predictors for coronary functional stenosis. **A** ROC curve for Carotid Plaque-RADS based on 1000 bootstrap resamples (gray lines), with the original curve in black (AUC = 0.81). **B** Comparative ROC curves for Carotid Plaque-RADS (AUC = 0.81), IPH volume (AUC = 0.74), LRNC volume (AUC = 0.63), total plaque volume (AUC = 0.70), and FRS (AUC = 0.59). Carotid Plaque-RADS, Carotid Plaque-Reporting and Data System; ROC, receiver operating characteristic; AUC, area under the curve; IPH, intraplaque hemorrhage; LRNC, lipid-rich necrotic core; FRS, Framingham risk score

**Fig. 4:**
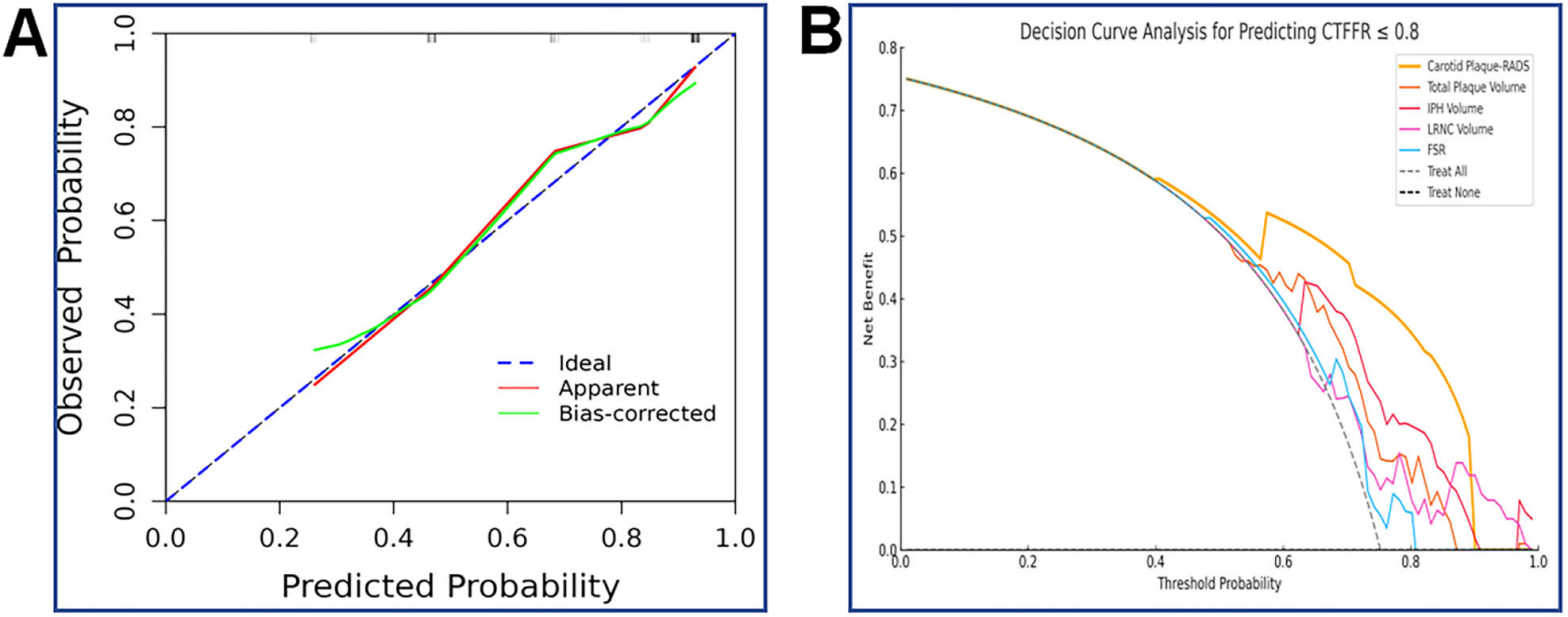
Calibration and decision curve analysis for Carotid Plaque-RADS and other predictors. **A** Calibration curve for Carotid Plaque-RADS using 1000 bootstrap resamples. The apparent (red) and bias-corrected (green) curves show model calibration, while the ideal (blue) line represents perfect agreement. **B** Decision curve analysis comparing net clinical benefit across a range of threshold probabilities for Carotid Plaque-RADS and the other predictors. Carotid Plaque-RADS, Carotid Plaque-Reporting and Data System; IPH, intraplaque hemorrhage; LRNC, lipid-rich necrotic core; FRS, Framingham risk score

The Carotid Plaque-RADS effectively predicted functional stenosis, with an AUC of 0.81 (95% CI: 0.71–0.90), accuracy of 81% (95% CI: 0.73–0.88), sensitivity of 85% (95% CI: 0.77–0.93), and specificity of 68% (95% CI: 0.49–0.86) at an optimal cutoff of 3b (Table 4). Figure 3A shows the ROC curve and AUC distribution for Carotid Plaque-RADS based on 1000 bootstrap resamples, yielding a mean AUC of 0.809 (95% CI: 0.71–0.89), indicating good model stability. The calibration curve (Fig. 4A) showed good agreement between the theoretical and actual outcomes. The Hosmer–Lemeshow test achieved a *p*-value of 0.43, suggesting a strong calibration. Using CAD-RADS ≥ 3 as the anatomical endpoint, Carotid Plaque-RADS demonstrated good discriminatory ability, with an AUC of 0.72 and a bootstrap mean AUC of 0.72 (95% CI: 0.60–0.83).

**Table 4:** Diagnostic performance of Carotid Plaque-RADS

AUC (95% CI)	Accuracy (95% CI)	Sensitivity (95% CI)	Specificity (95% CI)	PPV (95% CI)	NPV (95% CI)	Cutoff
0.81 (0.71–0.90)	0.81 (0.73–0.88)	0.85 (0.77–0.93)	0.68 (0.49–0.86)	0.89 (0.81–0.96)	0.60 (0.42–0.78)	3b

*AUC* area under the curve, *PPV* positive predictive value, *NPV* negative predictive value, *CI* confidence intervals, *RADS* Reporting and Data System

### Comparison of ROC curves for different predictors

The ROC curves for different predictors, including Carotid Plaque-RADS, total plaque volume, IPH volume and LRNC volume, and FRS, are shown in Fig. 3B. Carotid Plaque-RADS exhibited the highest AUC, significantly outperforming total plaque volume, IPH volume, and LRNC volume (*p *= 0.04, 0.01, and < 0.01, respectively). The largest AUC difference was observed between Carotid Plaque-RADS and FRS (ΔAUC = 0.21, *p* < 0.01), highlighting the superior predictive ability of Carotid Plaque-RADS over traditional risk scoring systems. Relevant results are listed in Table 5.

**Table 5:** Comparison of AUC differences between carotid Plaque-RADS and other predictors

Variable	AUC difference	SE	*Z*	95% CI	*p*-value
Carotid Plaque-RADS/IPH volume	0.07	0.31	2.65	0.01–0.11	0.01^*^
Carotid Plaque-RADS/LRNC volume	0.17	0.32	2.64	0.04–0.31	< 0.01^**^
Carotid Plaque-RADS/Total plaque volume	0.11	0.33	2.01	0.03–0.22	0.04^*^
Carotid Plaque-RADS/FRS	0.21	0.34	2.70	0.06–0.37	< 0.01^**^

*AUC* Area under the curve, *SE* standard error, *IPH* intraplaque hemorrhage, *LRNC* lipid-rich necrotic core, *FRS* Framingham risk score,* RADS* Reporting and Data System

* *p* < 0.05, ** *p* < 0.01

### DCA

The DCA for different predictors is shown in Fig. 4B. Carotid Plaque-RADS demonstrated the highest net benefit across most risk thresholds, further supporting its clinical utility in identifying patients with functionally significant stenosis.

## Discussion

This study showed that Carotid Plaque-RADS can predict coronary functional stenosis in candidates for carotid revascularization. Taken together, plaque characteristics, rather than stenosis severity alone, provide crucial insights into systemic atherosclerotic burden and coronary functional impairment. In clinical practice, patients with carotid atherosclerotic plaques undergo preoperative vascular evaluation to determine the need for carotid revascularization. Traditionally, surgical candidacy has been primarily decided based on the luminal stenosis severity. Revascularization is generally recommended for symptomatic patients with stenosis > 50% or for asymptomatic patients with stenosis > 70% [25, 26]. These criteria form the basis of most guideline-directed management strategies. However, growing evidence suggests that Carotid Plaque composition provides additional prognostic information [27, 28], consistent with our findings.

Patients with functionally significant coronary stenosis exhibited more severe carotid stenosis, larger plaque volumes, and a higher prevalence of calcification than those with normal coronary function. These findings are consistent with those of earlier studies showing a strong association between carotid atherosclerosis and CAD. According to the report by Naghavi et al and Fayad et al, Carotid Plaque features crucially predict adverse cardiovascular outcomes, including myocardial infarction and stroke [29–31]. IPH, a known marker of plaque vulnerability, destabilizes the plaque and increases the risk of rupture in both carotid and coronary arteries [11, 32]. LRNC is a well-established feature of vulnerable plaques and a key predictor of plaque rupture and clinical events [30, 32]. The role of calcification in atherosclerosis remains controversial; however, certain types, such as rim calcification, may increase the risk of rupture and ischemic complications [31].

Carotid Plaque-RADS emerged as an independent predictor of coronary functional stenosis, which further supports the clinical utility of this system in identifying high-risk patients. To account for the heterogeneity of intermediate-risk plaques, the original sub-classification of Carotid Plaque-RADS 3 into 3a, 3b, and 3c was preserved. In contrast, Plaque-RADS 4 lesions were analyzed collectively as a single high-risk category to avoid statistical bias arising from small subgroup sample sizes. Correlation analysis revealed that the Carotid Plaque-RADS score had the strongest association with coronary functional stenosis (*ρ* = 0.51, *p* < 0.01), highlighting the crucial function of Carotid Plaque-RADS in characterizing high-risk atherosclerotic features that correlate with functional impairment. Based on univariate analysis, Carotid Plaque-RADS was significantly associated with functional stenosis (OR: 2.47, *p* < 0.01), an association that remained significant after adjusting for confounders (OR: 2.35, *p* < 0.01). Carotid Plaque-RADS categorizes plaques based on structural features and vulnerability, enabling distinction between patients with and without functional coronary stenosis. These results highlight the value of evaluating Carotid Plaque characteristics as part of a comprehensive risk assessment.

Comparative analysis showed that Carotid Plaque-RADS demonstrated the highest diagnostic accuracy for identifying coronary functional stenosis (AUC = 0.81), outperforming IPH volume (AUC = 0.74), LRNC volume (AUC = 0.63), total plaque volume (AUC = 0.70), and FRS (AUC = 0.59). Compared with traditional risk scores, which primarily rely on demographic and clinical parameters, Carotid Plaque-RADS demonstrated superior diagnostic performance, while the FRS remains a cornerstone for predicting cardiovascular risk [33]. Additionally, although imaging biomarkers, such as IPH, LRNC, and calcification, were associated with functional stenosis in the univariate analysis, they failed to demonstrate any significance in the multivariate models. This supports the use of Carotid Plaque-RADS, which incorporates these features into a comprehensive scoring system for more accurate coronary risk assessment. In addition to predicting coronary functional stenosis, Carotid Plaque-RADS was moderately correlated with higher CAD-RADS grades in our cohort (*ρ* = 0.37, *p* < 0.01). Furthermore, it demonstrated good discrimination for identifying at least moderate coronary anatomical stenosis (CAD-RADS ≥ 3), with an AUC of 0.72 (95% CI: 0.60–0.83). These findings provide anatomical cross-validation of the systemic atherosclerotic burden captured by Carotid Plaque-RADS and complement the functional insights obtained from CT-FFR.

A cutoff at Carotid Plaque-RADS 3b yielded the highest diagnostic performance. Recent studies suggest the utility of HR-MRI-based Carotid Plaque-RADS for stroke risk stratification. A Carotid Plaque-RADS score ≥ 3 was an independent predictor of stroke [15], and Carotid Plaque-RADS has been shown to predict symptomatology in patients with carotid stenosis [17]. In a clinical cohort of patients with embolic stroke of undetermined source, a modified CTA-based Carotid Plaque-RADS classification showed that high-risk subtypes were more frequent on the stroke-ipsilateral side, supporting its utility for identifying potentially culprit carotid plaques [34]. These findings are consistent with our results and underscore the utility of Carotid Plaque-RADS as an imaging-based stratification tool and a noninvasive surrogate marker for identifying patients at an elevated risk of coronary ischemia. In candidates for carotid revascularization, the Carotid Plaque-RADS threshold of 3b helps identify individuals who may benefit from further coronary functional assessment, thereby improving perioperative cardiovascular risk management and guiding personalized treatment strategies. Incorporating Carotid Plaque-RADS into routine carotid imaging protocols could enhance comprehensive cardiovascular risk assessment.

DCA further supported the clinical utility of Carotid Plaque-RADS (Fig. 3), which consistently demonstrated the highest net benefit across various threshold probabilities, particularly in the intermediate-threshold range where most diagnostic uncertainties tend to occur. The Carotid Plaque-RADS curve did not intersect the “treat-all” or “treat-none” strategies, reinforcing its potential in improving clinical outcomes while minimizing unnecessary interventions. These findings align with its superior AUC and independent predictive value, supporting its incorporation into routine risk assessment pathways for patients with carotid atherosclerosis.

Carotid Plaque-RADS also demonstrated good calibration, as indicated by a non-significant Hosmer–Lemeshow test (*p* = 0.43), reflecting close agreement between predicted and observed outcomes. Together with its strong diagnostic accuracy, these findings underscore the reliability and practical value of Carotid Plaque-RADS for clinical decision-making, helping to prioritize patients most likely to benefit from further diagnostic evaluation or therapeutic interventions.

Beyond diagnostic performance, the feasibility of implementing Carotid Plaque-RADS in routine clinical practice is also an important consideration. The HR-MRI protocol required for Plaque-RADS evaluation can be completed within a reasonable acquisition time (20–30 min), as it relies on standard Carotid Plaque imaging sequences commonly used in vascular assessment. Furthermore, although the application of the Plaque-RADS system requires basic training to ensure consistent identification of plaque components and correct use of classification criteria, its structured design facilitates rapid learning and promotes reproducibility among readers. Collectively, these features support the practical feasibility of integrating Carotid Plaque-RADS into routine carotid imaging workflows.

This study has some limitations. First, the retrospective design and relatively small sample size may limit generalizability. Second, the use of two different MRI systems may have introduced variability in image quality; future studies employing standardized equipment are warranted. Third, no direct comparison with invasive coronary angiography, which is considered the gold standard test for evaluating coronary stenosis, was performed. Validation against angiography should be included in future research. Finally, the absence of longitudinal follow-up prevented assessment of plaque progression. Prospective studies with larger cohorts and long-term follow-up are warranted.

In conclusion, Carotid Plaque-RADS is an effective tool for predicting functionally significant coronary stenosis in patients being considered for carotid revascularization. A cutoff of Plaque-RADS 3b provides high diagnostic accuracy and helps identify patients at increased risk of coronary ischemia. By outperforming traditional cardiovascular risk factors and providing a structured assessment of Carotid Plaque characteristics, Carotid Plaque-RADS has the potential to be incorporated into routine imaging workflows to support clinical decision-making in patients with carotid atherosclerosis.

## Supplementary information

ELECTRONIC SUPPLEMENTARY MATERIAL

## Data Availability

The datasets generated and/or analyzed during the current study are not publicly available due to institutional and patient.

## Abbreviations

AUC: Area under the curve
CAD: Coronary artery disease
CCTA: Coronary computed tomography angiography
CE-T1WI: Contrast-enhanced T1-weighted imaging
CI: Confidence interval
CT-FFR: Computed tomography-derived fractional flow reserve
DCA: Decision curve analysis
FRS: Framingham risk score
HDL-C: High-density lipoprotein cholesterol
HR-MRI: High-resolution magnetic resonance imaging
IPH: Intraplaque hemorrhage
LRNC: Lipid-rich necrotic core
MWT: Maximum wall thickness
OR: Odds ratio
RADS: Reporting and Data System
ROC: Receiver operating characteristic
T1WI: T1-weighted imaging
T2WI: T2-weighted imaging
TC: Total cholesterol
TOF: Time-of-flight
