# Supplementary material for: Carotid Plaque-RADS improves preoperative coronary risk stratification in candidates for carotid revascularization
Source: Insights Imaging. 2026 Jan 26;17:18. doi: 10.1186/s13244-025-02188-y (PMC12834872; doi:10.1186/s13244-025-02188-y)
Supplement: Supplementary file 1 — ELECTRONIC SUPPLEMENTARY MATERIAL [file 13244_2025_2188_MOESM1_ESM.pdf]

Carotid Plaque-RADS improves preoperative coronary risk stratification in candidates for carotid revascularization

ELECTRONIC SUPPLEMENTARY MATERIAL

Insights Imaging (2025) Qin R, Zheng C, Zhang Y, et al.

Scanner	Sequence	TR (ms)	TE (ms)	Matrix	Notes
uPMR790	T1WI	800	14.52	220×220	Parallel imaging (acceleration factor=2)
uPMR790	T2WI	2000	198.44	220×220	Parallel imaging (acceleration factor=2)
uPMR790	3D-TOF	17.5	4	220×220	Parallel imaging (acceleration factor=2)
uPMR790	CE-T1WI	800	14.52	220×220	Gd-DTPA-BMA 0.1 mmol/kg @2.5 mL/s
Siemens Prisma	T1WI	425	20.5	220×220	Parallel imaging (acceleration factor=2)
Siemens Prisma	T2WI	2500	231.3	220×220	Parallel imaging (acceleration factor=2)
Siemens Prisma	3D-TOF	15	3.4	220×220	Parallel imaging (acceleration factor=2)
Siemens Prisma	CE-T1WI	425	20.5	220×220	Gd-DTPA-BMA 0.1 mmol/kg @2.5 mL/s

Imaging Parameters
